# Electrical stimulation: a potential alternative to positively impact cerebral health?

**DOI:** 10.3389/fphys.2024.1464326

**Published:** 2024-09-20

**Authors:** Maël Descollonges, Rémi Chaney, Philippe Garnier, Anne Prigent-Tessier, Julien V. Brugniaux, Gaëlle Deley

**Affiliations:** ^1^ INSERM UMR 1093 – Laboratoire CAPS, « Cognition, Action et Plasticité Sensorimotrice », Université de Bourgogne, Dijon, France; ^2^ Kurage, Lyon, France; ^3^ Département Génie Biologique, IUT, Dijon, France; ^4^ INSERM UMR 1300 – Laboratoire HP2, University Grenoble Alpes, CHU Grenoble Alpes, Grenoble, France

**Keywords:** neuromuscular electrical stimulation, functional electrical stimulation, cerebral blood flow, neuronal activity, cognition, neuroplasticity, humoral pathway

## Abstract

An increasing body of evidence confirms the effectiveness of physical exercise (PE) in promoting brain health by preventing age-related cognitive decline and reducing the risk of neurodegenerative diseases. The benefits of PE are attributed to neuroplasticity processes which have been reported to enhance cerebral health. However, moderate to high-intensity PE is necessary to induce these responses and these intensities cannot always be achieved especially by people with physical limitations. As a countermeasure, electrical stimulation (ES) offers several benefits, particularly for improving physical functions, for various neurological diseases. This review aims to provide an overview of key mechanisms that could contribute to the enhancement in brain health in response to ES-induced exercise, including increases in cerebral blood flow, neuronal activity, and humoral pathways. This narrative review also focuses on the effects of ES protocols, applied to both humans and animals, on cognition. Despite a certain paucity of research when compared to the more classical aerobic exercise, it seems that ES could be of interest for improving cerebral health, particularly in people who have difficulty engaging in voluntary exercise.

## Highlights

- Electrical stimulation (ES) is known to improved physical functions in healthy participants or people in rehabilitation.
- Mechanistically, this modality of exercise is associated with an increase in cerebrovascular function, neuronal activity, the release of neurotrophins and exerkines, eventually contributing to cerebral health.
- ES exercises could be of interest for improving cognition.

## 1 Introduction

Physical exercise (PE) is an essential part of a healthy lifestyle and is widely acknowledged as the most potent non-pharmacological approach to improve both physical and cognitive wellbeing (Hötting and Röder, 2013). Numerous studies have highlighted the positive effects of PE on cognition, coupled with cellular, molecular, functional, structural, and behavioral changes. For example, PE promotes hippocampal neurogenesis, synaptic plasticity, cerebral angiogenesis, as well as astrocyte and microglia plasticity (Hillman et al., 2008; Carlo Maria Di Liegro, 2019; El-Sayes et al., 2019). Mechanistically, these benefits are notably mediated by an elevation of the levels of cerebral brain-derived neurotrophic factor (BDNF), a molecule involved in neurogenesis and synaptic plasticity (Cefis et al., 2023) but also, insulin-like growth factor 1 (IGF-1), and vascular endothelial growth factor (VEGF). Although cerebral BDNF is known to be a determinant of plasticity (Cefis et al., 2023), the mechanisms underlying its upregulation in response to PE and the role of exercise modalities are not well-understood. Indeed, three mechanisms have been proposed (Cefis et al., 2023): (i) motor commands and afferent inputs from the periphery can increase neuronal activity in the brain and stimulate the expression of several growth factors (neuronal pathway), (ii) skeletal muscle can synthesize and secrete myokines, which enter the bloodstream and cross the blood-brain barrier to promote the expression of growth factors (endocrine pathway), and (iii) PE can increase cerebral blood flow (CBF), leading to an increase in shear stress and nitric oxide (NO) release, which can have multiple benefits for brain health (hemodynamic pathway). However, the relative contributions of these mechanisms remain unknown (Cefis et al., 2023). Nevertheless, it has been demonstrated that BDNF production by the cerebral vascular endothelium is both NO- and shear stress-dependent with the latter increasing during exercise (Monnier et al., 2017).

Recent data revealed that acute and chronic exercises performed at moderate intensities are associated with enhanced cognitive performances. However, people with physical limitations may not be able to perform such exercises. This predicament is particularly evident in spinal cord injuries (SCI) or stroke patients. Additionally, elderly individuals may grapple with obstacles to engage in active PE routines due to their physical frailty. Finally, various medical conditions, such as heart failure or chronic obstructive pulmonary disease (COPD) are associated with medical contraindications and exercise intolerance. Over the (recent) years, electrical stimulation (ES) has been proposed as a possible effective alternative to traditional voluntary physical exercise when the latter becomes compromised (Doucet et al., 2012; Maffiuletti et al., 2018). Indeed, it has been reported that ES can be used to preserve, restore or even improve physical and neuromsucular function. In addition, it is generally used in rehabilitation context, notably to improve muscle strength, increase range of motion, reduce inflammation, counter muscular atrophy and weakness, and reduce muscular pain.

Although it has received less attention than physical outcomes in the literature, a recent study evidenced that ES could be of great interest to improve cerebral health (Descollonges et al., 2024). In this narrative review, we have opted to present results in both humans and animals, since the latter allows us to approach mechanisms that would otherwise remain untouched while focusing solely on human studies, to explore the impact of ES on the cerebral and mental health.

## 2 Main text

### 2.1 Electrical stimulation

#### 2.1.1 Definition

Electrical stimulation is a technique used to elicit muscle contractions by applying electrical impulses to the skin above the muscle or along the pathway of a superficial nerve (Deley et al., 2015; Blazevich et al., 2021). ES is now an integral part of clinical settings, especially in neurological rehabilitation, proving effective for patients with impaired voluntary contractions, such as those with spinal cord injuries or stroke (Deley et al., 2015; Stein et al., 2015). In addition, ES aims to preserve, restore, or even enhance physical and neuromuscular functions, with the primary goals of improving muscle strength, increasing one’s range of motion, reducing inflammation, counteracting muscle atrophy and weakness, and alleviating muscle pain (Doucet et al., 2012). Regular use over weeks can induce neural adaptations, yield positive health outcomes, and enhance both quality of life and wellbeing, in turns, addressing both clinical and non-clinical objectives (Blazevich et al., 2021).

#### 2.1.2 Models of electrical stimulation

Among ES techniques, two primary modalities can be distinguished: Neuromuscular Electrical Stimulation (NMES) and Functional Electrical Stimulation (FES) (Maffiuletti et al., 2018). Transcutaneous electrical nerve stimulation (TENS), a third technique employing a low-intensity and continuous electrical current on cutaneous nerve fibers, is mainly used for treating chronic and acute pain but falls outside the scope of this review. NMES is traditionally applied under isometric tetanic conditions, employing intermittent or high-intensity electrical stimuli to generate robust muscle contractions (Maffiuletti et al., 2018). This technique can be directed either to the muscle belly (myostimulation) or along the nerve pathway (neurostimulation). On another hand, FES involves applying moderate-intensity and cyclic electrical stimulations to selected muscles (Maffiuletti et al., 2018). The following sections focus on NMES, while we decided to dedicate the last part of this review to FES, even if it has recevied less attention to date.

### 2.2 Effects of NMES on the brain

As previously mentionned, three primary mechanisms associated with the cerebral benefits induced by PE have been evidenced: heightened neuronal activity, increased cerebral perfusion, and the release of exerkines from peripheral tissues (Cefis et al., 2023). After having delved into the influence of NMES protocols on these mechanisms in both human and non-human subjects, the following section will seek to describe its cognitive and behavioral effects.

#### 2.2.1 Potential mechanisms involved in NMES induced cerebral benefits

**FIGURE 1:**
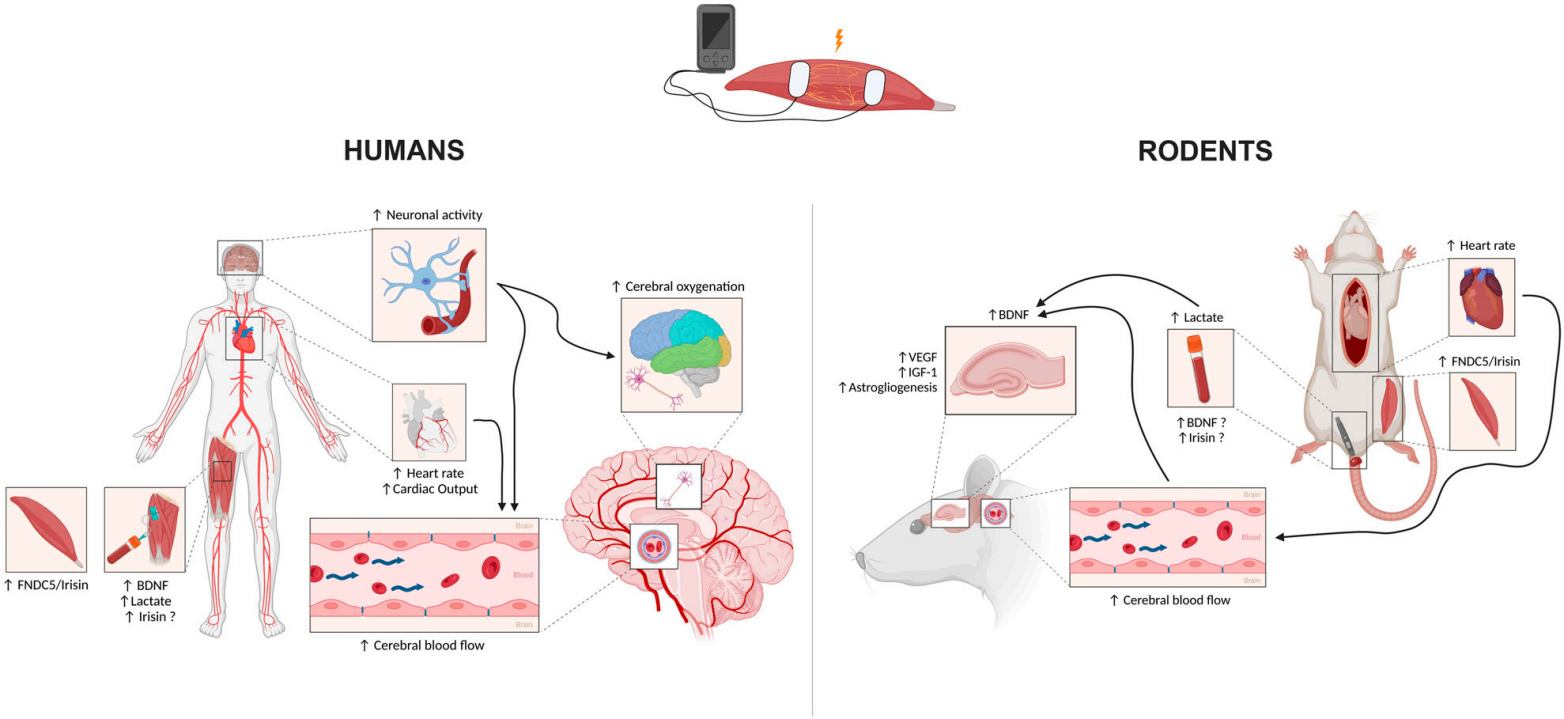
Potential mechanisms underlying the positive effects of electrical stimulation on cerebral health in both humans (left) and rodents (right). Black arrows pointing upwards represent an increase. Black arrows running between mechanisms represent potential links. BDNF: Brain-Derived Neurotrophic Factor; FNDC5: Fibronectin type III domain-containing protein 5. Created with www.BioRender.com.

Potential mechanisms involved in NMES-induced cerebral benefits are summarized in Figure 1.

##### 2.2.1.1 Effects on neuronal activity

It is widely recognized that neuronal activity increases during either NMES or FES sessions on the upper or lower limbs in both healthy individuals and patients (Smith et al., 2003; Blickenstorfer et al., 2009; Chipchase et al., 2011; Joa et al., 2012; Wegrzyk et al., 2017). Both activate predominantly regions associated with sensorimotor control, the thalamus, and the cerebellum. The observed increase in neuronal activity within these regions, even in the absence of motor commands, could potentially stem from the activation of neuromuscular spindles in response to electrical stimulation (Francis et al., 2000) or the stimulation of skin afferences (Allison et al., 1991).

Several studies have indeed reported an elevation of H+ ions and a depletion of adenosine triphosphate (ATP) during NMES, which should lead to the activation of the metaboreflex to regulate heart rate (HR) and ventilation. However, the variations in HR and ventilation during NMES are minimal. Additionally, afferent signals from the metaboreflex primarily project to the brainstem, and to our knowledge, no imaging studies have reported an increase in neuronal activity in this region (Michelini et al., 2015). Furthermore, no change in c-fos hippocampal expression, a marker of calcium influx-induced neuronal activity, was observed in mice (1 session, 100 Hz, 0.1 ms, 40 contractions, sciatic nerve) (Gardner et al., 2020) and rats subjected to acute NMES (1 session, 100 Hz, 400 μs, 7s-ON/14s-OFF, 6–20 mA, lower limbs) (Chaney et al., 2024). In contrast to NMES, traditional treadmill PE has been shown to increase c-fos in both the sensorimotor cortex and cognitive regions (Cefis et al., 2019). This discrepancy between PE and NMES could be attributed to the absence of locomotion, coordination, intentions and spatial engagement during NMES, which are crucial for hippocampal activation (Eichenbaum, 2017; Joshi et al., 2023).

##### 2.2.1.2 Effects on hemodynamic parameters

There is limited research on the effect of NMES protocols on CBF modulations. One study showed regional differences in the CBF response with an increase in the internal carotid artery (ICA) but not in the vertebral artery (VA), in response to NMES (1 session, 20 min, 4 Hz, 250 μs, maximal tolerable intensity, whole lower limbs) (Ando et al., 2021). These findings were recently corroborated by Descollonges et al. (2024) who observed that a single NMES session increases blood flow velocity through the middle (MCA) but not the posterior cerebral artery (PCA) (1 session, 25 min, 40 Hz, 400 μs, 6s-ON/6s-OFF, maximal tolerable intensity, quadriceps muscles) (Descollonges et al., 2024). These results align with a study in rats showing that a single session of NMES applied on lumbar nerve roots to contracted hindlimb muscles, increased CBF in the sensorimotor cortex (1 session, 30 min, 100 Hz, 200 μs, 6s-ON/3s-OFF, 2.5- to 5- fold the motor threshold, lumbar nerve roots) (Chaney et al., 2022). However, the authors also reported no increase of CBF in cerebral regions involved in cognition such as the prefrontal cortex and hippocampus evaluated by the phosphorylation level of the endothelial nitric oxide synthase (eNOS) (Chaney et al., 2022), which seems logical since neither of these areas is primarily supplied by the ICA or MCA.

To match the increase in metabolic activity, cerebral oxygenation increases in parallel with the increase in flow (Perrey, 2008; Smith and Ainslie, 2017). Accordingly, previous studies observed increases in cerebral oxygenation of the sensorimotor cortex (1 session, 30 Hz, 200 μs, wrist extensors muscles) (Muthalib et al., 2015) and left prefrontal cortex during NMES (Descollonges et al., 2024).

At the cardiovascular level, studies have reported a significant increase in cardiac output throughout NMES, which in turns would translate into an increase in CBF (Jordan and Sheel, 2017; Smith and Ainslie, 2017). In addition, to meet the resting metabolic demand of neuronal activity, approximately 15%–20% of the CBF is derived from cardiac output, the latter increase being directly dependent on the muscle mass involved in PE (Montalvo et al., 2022). Hence, the muscle mass emerges as a pivotal factor influencing the observed increase in CBF. This, however, needs to be confirmed by future research, especially using FES where, by design, a greater muscle masses is involved.

##### 2.2.1.3 Effects on exerkines release

It is well-established that peripheral tissues such as skeletal muscle release exerkines, into the bloodstream, thereby inducing improvements in cerebral health (Chow et al., 2020; Cefis et al., 2023).

##### 2.2.1.4 Lactate

Currently, lactate is the exerkine that has received the most attention in NMES studies. Notably, it has been documented that lactate can cross the brain-blood-barrier (BBB) (Pellerin et al., 1998; Proia et al., 2016) and is intricately involved in neuroplastic processes such as hippocampal neurogenesis and synaptic plasticity (Yang et al., 2014; Lev-Vachnish et al., 2019). For example, lactate can cross the BBB via its monocarboxylate transporters (MCTs) (Proia et al., 2016). Inhibition of this transporter prevents the improvement of spatial memory and synaptic plasticity in response to exercise (El Hayek et al., 2019). Futhermore, investigations have confirmed that an acute PE session leads to an immediate increase in cerebral lactate concentration in rats. In humans as well, acute PE raises lactate levels in the cerebrospinal fluid (Bisgard et al., 1978). Studies have reported elevated blood lactate levels following a NMES session targeting the quadriceps muscles in both humans and rats (Chaney et al., 2024). Interestingly, we recently observed positive correlations between lactate production and either Stroop Task improvement in healthy humans or hippocampal BDNF levels in rats following an NMES session (1 session, 100 Hz, 400 μs, 7s-ON/14s-OFF, 6–20 mA in rats, maximal tolerable intensity in humans, quadriceps muscles) (Chaney et al., 2024). The mechanisms through which lactate can influence brain health are multiple. They include the transport of lactate into neurons via MCT2, which increases the activity of the deacetylase sirtuin 1 (SIRT1). This increase can, in turn, enhance the transcriptional activity of PGC-1α, leading to the induction of BDNF (El Hayek et al., 2019) Interestingly, ES in rats increases hippocampal expression of SIRT1 (Chaney et al., 2024), suggesting a SIRT1-dependent mechanism in the beneficial effects of lactate in the context of ES.

When considered together, these findings strongly imply that lactate plays a pivotal role as an essential exerkine mediating the positive effects of NMES on cerebral health.

##### 2.2.1.5 Cathepsin-B

As mentioned in a recent review (Cefis et al., 2023), cathepsin B (CTSB) is thought to play an important role in brain health, notably through its effect on BDNF and neurogenesis (Moon et al., 2016). Although studies that have examined the effects of NMES protocols on cathepsin-B are poor, a recent study showed that NMES applied at stimulation frequencies of 20 Hz induced significantly greater increases in serum cathepsin-B levels than evoked stimulation at 4 or 80 Hz or the control condition in healthy young subjects (1 session, 20 min, stimulation duration varied between 250 ms (4 Hz condition), 50 ms (20 Hz condition) and 12.5 ms (80 Hz condition), 100 μs, entire lower limbs) (Nishikawa et al., 2024). However, to date, only one study has investigated CTSB after NMES application, and it would be interesting in future to examine the effects of an NMES- or FES-based intervention on CTSB while measuring cognitive performance, in order to verify whether the link found between conventional PE and CTSB is also found with this intervention.

##### 2.2.1.6 FNDC5/Irisin

Recent studies have provided compelling evidence indicating that the activation of the FNDC5/Irisin (Fibronectin type III domain-containing protein 5) pathway in skeletal muscle is involved in PE-induced cerebral plasticity (Bao et al., 2022). For instance, it has been reported increases circulating irisin after whole-body NMES combined with HIIT-type exercise in obese participants (1 session, 25 min, 85 Hz, 400 μs, 10s-ON/2s-OFF, whole body) (Ghalamsiah and Nourshahi, 2023). Interestingly, rodent data strongly suggest that fast-twitch fibers could be the key contributor to the surge in circulating Irisin levels after PE (Leger et al., 2024). This could be particularly relevant for disabled populations where the percentage of fast-type fibers is usually elevated (Schaufelberger et al., 1995; Toth et al., 2016).

On another hand, the effects of NMES on FNDC5/Irisin are less clear since an elevation in muscular concentration of FNDC5/Irisin has been reported in both humans and rodents after NMES (Maekawa et al., 2018; Petrie et al., 2020; Chaney et al., 2024), but without any changes in circulating irisin, at least 3, 4 and 24 h after an acute NMES protocol in rats (Maekawa et al., 2018; Chaney et al., 2024). Similarly, no changes were observed in hippocampal FNDC5/Irisin levels (Chaney et al., 2024). These conflicting data underline the necessity for further studies to clarify the impact of NMES on the production of circulating irisin.

##### 2.2.1.7 Effects on brain-derived neurotrophic factor (BDNF)

Given the inherent challenges of *in vivo* quantitfication of BDNF in the brain, human studies are resorting to assessing circulating BDNF levels (Walsh and Tschakovsky, 2018). Despite being considered a potential biomarker of cerebral health in humans, it remains unclear whether circulating BDNF can cross the blood brain barrier (BBB) and could, therefore, really be considered a surrogate for brain levels of BDNF (Wu and Pardridge, 1999).

Over the last decade, it has been reported that NMES protocols have the potential to induce an increase in circulating levels of BDNF both in humans (Wahl et al., 2015; Miyamoto et al., 2018a; b; Kimura et al., 2019; Nishikawa et al., 2021; 2024; Ghalamsiah and Nourshahi, 2023) (Table 1) and in rodents (Ke et al., 2011; Lin et al., 2015b; Dalise et al., 2017; Maekawa et al., 2018; Chaney et al., 2024) (Table 2). However, some degree of discrepancy remains since few studies failed to observed increases in circulating BDNF levels in Parkinson’s patients (Fiorilli et al., 2021) or in rats following NMES (Chaney et al., 2024). Interestingly, Nishikawa et al. (2022) showed that bilateral NMES of the entire lower limbs in healthy participants resulted in significantly higher serum BDNF concentration compared to stimulation of the quadriceps alone, indicating that circulating BDNF levels increase depending on engaged muscle mass (Nishikawa et al., 2022). Recently, same authors reported that NMES at 20 Hz induced significantly larger increases in BDNF serum levels than stimulation at 4 or 80 Hz or the control condition in healthy young adults indicating that BDNF levels increase depending also of the frequency of stimulation (Nishikawa et al., 2024). Collectively, most studies indicate that NMES stimulates the production of circulating BDNF, consistent with recent data obtained in mice showing that skeletal muscles can secrete BDNF into the bloodstream to regulate glucose homeostasis (Fulgenzi et al., 2020). Indeed, transgenic mice with a deletion of the bdnf gene in skeletal muscles exhibit reduced circulating levels of BDNF (Fulgenzi et al., 2020). Additionally, *ex vivo* electrical stimulation of the diaphragm induces the release of BDNF into the culture medium (Fulgenzi et al., 2020). However, whether muscle-secreted BDNF can have effects on the brain requires further research.

**TABLE 1:** Electrical stimulation programs used to investigate the effects on BDNF expressions in humans.

Authors	Population	Number	Stimulation	Frequency	Wide pulse	Current amplitude	ON/OFF	Sessions duration	Stimulated muscles	Main findings
Ghalamsiah and Nourshahi (2023)	Overweight	13	NMES	85 Hz	400 μs	-	10 s ON - 2 s OFF	1 session (25 min/session)	Whole body	↑ BDNF (Serum) ↑ Irisin (Serum)
Nishikawa et al. (2022)	Able-bodied	12	NMES	20 Hz	100 μs	Max. tolerable	Ranged from 50 to 200 ms	1 session (23 min/session)	Quadriceps Triceps surae	↑ BDNF (Serum)
Fiorilli et al. (2021)	Parkinson’s patients	12	NMES	85 Hz	350 μs	Max. tolerable	4 s ON - 4 s OFF	1 session (20 min/session)	Quadriceps	No increase of BDNF (Serum)
Nishikawa et al. (2021)	Elderly	3	NMES	20 Hz	100 μs	4.85 mA	5 s ON - 10 s OFF	5 sessions/w during 8 weeks (23 min/session)	Quadriceps	↑ BDNF (Serum)
Kimura et al. (2019)	Able-bodied	11	NMES	20 Hz	50 μs	31.3 mA	4.5 s ON – 4.5 s OFF	1 session (20 min/session)	Quadriceps	↑ BDNF (Serum)
Miyamoto et al. (2018a)	Type II Diabete	14	NMES	4 Hz	200 μs	Max. tolerable	Unspecified	5 sessions/w during 8 weeks (40 min/session)	Gluteus Quadriceps HamstringsTriceps surae	↑ BDNF (Plasma)
Miyamoto et al. (2018b)	Able-bodied	13	NMES	4 Hz	250 μs	Max. tolerable	Unspecified	1 session (30 min/session)		
Wahl et al. (2015)	Able-bodied	13	FES-cycling vs NMES	60 Hz	400 μs	Max. tolerable	Continous for myostimulation	1 session/condition (60 min/session)	Lower limbs	↑ BDNF (Serum) only for FES condition

NMES: neuromuscular electrical stimulation; BDNF: Brain‐Derived Neurotrophic Factor; ON: time of contraction; OFF: resting time.

**TABLE 2:** Electrical stimulation programs used to investigate the effects on BDNF expressions in animal’s model.

Authors	Population	Stimulation	Frequency	Wide pulse	Current amplitude	ON/OFF	Sessions duration	Stimulated muscles	Main findings	Other results
Chaney et al. (2024)	Rats	NMES	100 Hz	400 μs	6 – 20 mA	7 s ON – 14 s OFF	1 session (80 contractions)	Quadriceps	↑ BDNF (hippocampus)	↑ FNDC5/irisin after 24 h (quadriceps) ↑ Lactate
Maekawa et al. (2018)	Rats	NMES	100 Hz	1000 μs	Motor Threshold	3 s ON - 7 s OFF	1 session (50 contractions)	Sciatic nerve	↑ BDNF protein/mARN (hippocampus)	↑ FNDC5/irisin (hippocampus) NMES doesn’t increase muscle BDNF and muscle FNDC5
Dalise et al. (2017)	Rats	NMES	50 Hz	150 μs	15 mA	5 s ON - 10 s OFF	5 sessions /w during 4 weeks	Brachial biceps Brachial triceps	↑ BDNF (Serum)	↑ ARNm BDNF (Hippocampus) for the low NMES dose ↑ Lactate
Lin et al. (2015)	Rats	FES	100 Hz	300 μs	Motor Threshold	0.15 ON – 0.6 s OFF	30 min/days during 2 weeks	Wrist extensors	↑ BDNF (hippocampus/prefrontal cortex)	↑ TrkB (hippocampus/prefrontal cortex)
Ke et al. (2011)	Rats	FES	100 Hz	300 μs	Motor Threshold	TA: 0.05 s ON MG: 0.15 s ON Both: 300 s OFF	7 days (30 min/days)	Tibialis Anterior Triceps surae	↑ BDNFm (Hippocampus/striatum)	BDNF levels ++ in striatum for FES group but for hippocampus voluntary group is more efficient

NMES: neuromuscular electrical stimulation; FES: functional electrical stimulation; BDNF: Brain‐Derived Neurotrophic Factor; FNDC5: Fibronectin type III, domain‐containing protein 5; TrkB: Tropomyosin receptor kinase B; ON: time of contraction; OFF: resting time.

#### 2.2.2 Cognitive and behavioral effects in response to NMES

There is no consensus regarding the effect a single bout of NMES on cognition. Indeed, some studies both in animal and human suggested no improvement in cognitive performance. For instance, in humans, neither the Stroop test nor the Wisconsin card task (assessment of cognitive flexibility and executive functions by requiring subjects to sort cards according to changing rules (color, shape, number)) (Miyamoto et al., 2018a), or a Go/No-Go task (reaction time task), were altered after a single NMES session with low frequencies (4–20 Hz, 250 μs) (Ando et al., 2024a; b; Sudo et al., 2024). However, a recent study reported reaction time improvements to the Go/No-Go task when NMES is combined with voluntary arm cranking (Ando et al., 2024a). In rodents, a study involving mice subjected to an acute sciatic nerve stimulation protocol (100 Hz, 0.1 ms, 40 contractions, 4-s ON/4-s OFF) revealed no improvement in performance in the Morris water maze, rotarod, and contextual fear conditioning test (Gardner et al., 2020). This lack of improvement was linked to an increase in astrogliogenesis without concurrent changes in hippocampal neurogenesis. On another hand, recent reports indicate that an acute isometric session of NMES applied to the quadriceps at both low (40 Hz) (Descollonges et al., 2024) or high frequencies (100 Hz) (Chaney et al., 2024) can enhance Stroop task scores and reduce anxiety in healthy participants. In these studies, a three-step Stroop task was performed and consisted of read the most color names (green, blue, yellow, and red) printed in black, name the most color patches, and state the most color words printed with inconsistent color ink for 45 s. However and while the literature on chronic use of NMES is even more scarce and limited to humans, it has been shown in advanced laryngeal cancer patients that 8 weeks of NMES did not demonstrate any advantages for anxiety, depression, or sleep quality (30 min/session, 2 sessions/week during 8 weeks, 2–100 Hz, maximal tolerable intensity) (Zhang et al., 2018). Nevertheless, if one wants to expand beyond the scope of this review, there is more literature suggesting that ES, including NMES, FES and Hybrid FES protocols, can be beneficial notably for the quality of life (Durmus et al., 2009; Descollonges et al., 2023; Ramezani et al., 2023). Lastly, a recent protocol study suggests evaluating the chronic effects of NMES on cognition and BDNF over 12 weeks in SCI patients, and this work should be followed closely in the future (Vints et al., 2024).

Taken together, these results are calling for more research on this topic, especially when considering the variety in stimulation parameters available from the literature thus far. Moreover, future clinical and preclinical studies are imperative to unravel the nuanced effects of NMES on cognitive function.

#### 2.2.3 The specific case of functional electrical stimulation (FES)

As mentionned previously, NMES alone might be insufficient to improve cognition but this intervention can potentiate the effect of other strategies (i.e., FES) acting directly on cognition thanks to its effects on neuroplasticity processes. FES involves a voluntary contribution and therefore could potentially potentiate the effects of NMES. This modality holds promises, notably as it can induce functional movements and engage a substantial muscle mass (Deley et al., 2015). Currently, two modalities of FES are employed to induce muscle contractions in the lower limbs, namely, FES-Cycling and FES-Rowing. Both involve electrical stimulation to the lower limb, while voluntarily exercising either with lower- (cycling) or upper-body (rowing) muscles, eventually generating a complete rowing movement for the latter (Deley et al., 2015; Ye et al., 2021). In recent years, FES techniques have emerged as compelling alternatives and complementary solutions to assist patients in generating voluntary movement of moderate to high intensity.

A distinction can be made between exercises that are performed solely with FES (called FES-induced) and those where FES is used as an aid (FES-assisted). Only few studies focused on the mechanisms involved in neuroplasticity following demonstrating elevated blood lactate levels (Gojda et al., 2019) in healthy participants and cerebral oxygenation (Lo et al., 2018) in stroke patients following a FES-assisted as well as increases in brain activity during FES-induced wrist movement (Joa et al., 2012). In the future, studies would need to explore other FES-induced putative mechanisms involved in neuropalsticity such as alterations in cerebral blood flow.

There is a clear paucity of data on the acute effect of FES on cognition and/or behavior, either in humans or in animals. On another hand, it has been reported, using a stimulation protocol aiming to emulate a walking pattern resembling an FES protocol (100 Hz, 0.3 ms, 3 × 10 min/day for 2 weeks, running model at a speed of 12 m/min) in a rat model of cerebral hypoperfusion (bilateral carotid occlusion), that FES effectively restores performance on the object recognition test and the Barnes maze, assessing memory function (Lin et al., 2015b). The authors highlighted that these positive effects on behavior were associated with an increase in BDNF and downstream signaling pathways in the hippocampus (Lin et al., 2015a). Additionally, the authors observed an increase in synaptic protein levels, along with enhanced survival of hippocampal neurons (Lin et al., 2015a. These promising outcomes align with similar findings on the impact of FES on BDNF expression in both hippocampus and striatum in a rat model of stroke (middle cerebral artery occlusion) (Lin et al., 2015a). Intriguingly, the elevation in cerebral BDNF was similar to what is observed following both voluntary and forced treadmill exercise (Ke et al., 2011).

In humans, 30 weeks of FES in spinal cord injury (SCI) patients showed long-term psychological improvement and an antidepressant effect (Donna, 1992). Additionally, if one recent study reported that FES-assisted cycling do not impact cognitive performance (Go/No-Go task) in healthy participants (Ando et al., 2024b) it has been demonstrated that this modality could induce moderate-to-large progress in cognitive processing speed (Pilutti et al., 2019) and reduce delirium (Parry et al., 2014). Mechanistically, since cerebral BDNF cannot be measured *in situ* in humans, one has to rely on indirect markers. For instance, it has been suggested that the muscle mass involved and the stimulation parameters play a critical role in the measured outcomes on cognition and/or behavior. The involvement of a large muscle mass would translate into an elevation in CBF as a result of increased neuronal activity, hypercapnia and increased cardiac output (Jordan and Sheel, 2017; Smith and Ainslie, 2017), as well as increases of blood levels of irisin and lactate, which might be involved in cerebral BDNF upregulation (Cefis et al., 2023). Thus, when compared to NMES, it could be hypothesized that FES might have a greater impact on physical performance or indeed cognition than NMES, due to the larger muscle mass involved during the exercise. Taken together and despite the limited array of studies in the literature, it seems that FES interventions represent a promising methodology to improve cognition.

## 3 Methodological considerations

Electrical stimulation may have certain limitations, the main ones being listed thereafter.

### 3.1 Muscle damage

According to the stimulation parameters use, muscle damage could manifest histologically by the apoptosis of muscle fibers, the infiltration of inflammatory cells, and the disruption of sarcomeric organization (Mackey et al., 2008). While muscle damage can have a positive effect on strength, it is important to note that it can also negatively affect the effect of exercise on the brain. The mechanisms by which PE induces muscle damage exceed the scope of this review and have been described elsewhere (Fouré and Gondin, 2021). In animals, for example, it has been demonstrated that muscle injury could promote neuroinflammation and impair hippocampus-dependent memory (Guéniot et al., 2020). In addition, recent results showed that heightened activations of neuroinflammatory processes can lead to alterations in synaptic plasticity and BDNF expression (Golia et al., 2019). Thus, controlling the impact of NMES protocols on muscle damage appears crucial when targeting cerebral health enhancement but also to ensure patient’s adherence to the training protocol.

### 3.2 Discomfort

On another hand, NMES is often associated with discomfort felt during the application of ES on the skin. This discomfort is even more pronounced in women and obese individuals (Maffiuletti, 2010), since adipose tissue acts as a capacitor, hindering the passage of current to the muscle tissue. Moreover, placing the electrodes on a motor point can reduce the sensation of discomfort and improve muscle activation (Gobbo et al., 2014). The size of the electrodes is also important: several experiments comparing different positionning configurations, reported higher tolerated intensities of stimulation (i.e., higher torque) and lower discomfort when using large electrodes (Maffiuletti et al., 2014; Barss et al., 2018). Indeed, with small electrodes, current density is higher and might produce a preferential excitation of small-diameter sensory fibers which are sensitive to current density in the dermo-epidermal junction (Mørch et al., 2011; Bergquist et al., 2017).

It is plausible that the perception of discomfort during ES affects its effects on the brain. For example, it has been reported that cortical activation during ES is correlated with the discomfort experienced by subjects. Thus, greater discomfort could induce a higher level of arousal and have short-term positive effects on cognition due to elevated arousal. Further studies would be interesting to evaluate the relationship between ES-induced discomfort, arousal levels, and cognition. On the other hand, ES can induce substantial muscle damage (as described in the manuscript) in the days following its application. The nociceptive nerve endings of skeletal muscles respond to the release of ATP due to sarcolemma permeabilization, muscle inflammation, pH variations, and muscle temperature changes. Various clinical and preclinical studies have highlighted the link between chronic pain, the emergence of anxiety-like behaviors, and impaired cognitive function. Therefore, it will be interesting in the future to evaluate cognition in the days following the application of ES. Moreover, it is also known that ES is perceived as less uncomfortable when combined with voluntary contraction. Hybrid FES may therefore be an interesting solution. Thus, it is therefore essential to carry out pre-conditioning and familiarization sessions, while modulating the intensity and/or force developed to reduce discomfort, muscle damage or fatigue.

## 4 Conclusion

Taken together, this review highlights the fact that ES has received little attention compared to aerobic exercise, but the available data suggest that ES could be of interest for improving cerebral health, particularly in people who cannot exercise voluntarily. Further studies are needed to confirm this postulate and elucidate the underlying mechanisms. In addition, stimulation protocols need to be optimized to reduce muscle damage and fatigue. It would, therefore, be appropriate to carry out comparative studies in humans and animals between conventional PE, NMES and FES.

## Abbreviations

ATP: Adenosine triphosphate; BBB: Brain blood barrier; CBF: Cerebral blood flow; COPD: Chronic obstructive pulmonary disease; eNOS: Endothelial nitric oxide synthase; ES: Electrical stimualtion; BDNF: Brain-derived neurotrophic factor; FES: Functional electrical stimulation; FNDC5: Fibronectin type III domain-containing protein 5; HIIT: High intensity interval training; ICA: Internal carotid artery; MCA: Middle cerebral artery; NMES: Neuromuscular electrical stimulation; NO: Nitric oxide; PCA: Posterior cerebral artery; PE: Physical exercise; SCI: Spinal cord injury; TENS: Transcutaneous electrical nerve stimulation; VA: vertebral artery.
